# Evaluation of a hybrid automatic planning solution for rectal cancer

**DOI:** 10.1186/s13014-022-02129-9

**Published:** 2022-10-13

**Authors:** Jiyou Peng, Lei Yu, Fan Xia, Kang Zhang, Zhen Zhang, Jiazhou Wang, Weigang Hu

**Affiliations:** 1 Department of Radiation Oncology, Fudan University Shanghai Cancer Center, Shanghai, China; 2 Department of Oncology, Shanghai Medical College Fudan University, Shanghai, China; 3 Shanghai Key Laboratory of Radiation Oncology, Shanghai, China; 4 United Imaging Healthcare, Shanghai, China

**Keywords:** Automatic planning, Hybrid planning, Script-based planning, Knowledge-based planning, Rectal cancer

## Abstract

**Background:**

Script-based planning and knowledge-based planning are two kinds of automatic planning solutions. Hybrid automatic planning may integrate the advantages of both solutions and provide a more robust automatic planning solution in the clinic. In this study, we evaluated and compared a commercially available hybrid planning solution with manual planning and script-based planning.

**Methods:**

In total, 51 rectal cancer patients in our institution were enrolled in this study. Each patient generated 7 plans: one clinically accepted manual plan (${plan}_{manual}$), three script-based plans and three hybrid plans generated with the volumetric-modulated arc therapy technique and 3 different clinical goal settings: easy, moderate and hard (${plan}_{hybrid}^{easy}$, ${plan}_{hybrid}^{moderate}$, ${plan}_{hybrid}^{hard}$, ${plan}_{script}^{easy}$, ${plan}_{script}^{moderate}$ and ${plan}_{script}^{hard}$). Planning goals included planning target volume (PTV) D_max_, bladder D_mean_ and femur head D_mean_. The PTV prescription was the same (50.00 Gy) for the 3 goal settings. The hard setting required a lower PTV D_max_ and stricter organ at risk (OAR) dose, while the easy setting was the opposite. Plans were compared using dose metrics and plan quality metric (PQM) scores, including bladder D_15_ and D_50_, left and right femur head D_25_ and D_40_, PTV D_2_, D_98_, CI (conformity index) and HI (homogeneity index).

**Results:**

Compared to manual planning, hybrid planning with all settings significantly reduced the OAR dose (*p* < 0.05, paired t-test or Wilcoxon signed rank test) for all dose-volume indices, except D_25_ of the left femur head. For script-based planning, ${plan}_{script}^{easy}$ significantly increased the OAR dose for the femur head and D_2_ and the PTV homogeneity index (*p* < 0.05, paired t-test or Wilcoxon signed rank test). Meanwhile, the maximum dose of the PTV was largely increased with hard script-based planning (D2 = 56.06 ± 7.57 Gy). For all three settings, the comparison of PQM between hybrid planning and script-based planning showed significant differences, except for D_25_ of the left femur head and PTV D_2_. The total PQM showed that hybrid planning could provide a better and more robust plan quality than script-based planning.

**Conclusions:**

The hybrid planning solution was manual-planning comparable for rectal cancer. Hybrid planning can provide a better and more robust plan quality than script-based planning.

**Supplementary Information:**

The online version contains supplementary material available at 10.1186/s13014-022-02129-9.

## Background

Intensity-modulated radiation therapy (IMRT) has been a commonly used method for radiotherapy [1]. The basic features of IMRT contribute to its advantages: better planning target volume coverage and sparing of OAR [2]. However, modern radiotherapy treatment planning is complex and time-consuming, which requires considerable trial-and-error to obtain quality plans, even for experienced physicists [3–6]. The demand for labor-saving and optimal plans promoted the emergence of automatic planning both in research and the clinic.

Script-based planning is a typical automatic planning solution. By modeling the reasoning process based on complex and extensive human knowledge, script-based planning simulates steps of the manual planning process through ‘if–then’ binary actions [7]. Studies of in-house developed script-based planning have been booming for years [8, 9], but their in-house characteristics and third-party coding languages have limited their implementation in the broader community and in radiotherapy clinics [10]. Vendors of modern TPS have provided scripting solutions, one of which is a scripting programming interface to enable scripting functions by users’ inputs, such as ESAPI [11] (Varian Medical System, Palo Alto, California), and another is enhanced integration, such as AutoPlanning [12] (Philips Radiation Oncology Systems, Fitchburg, Wisconsin), a product as an optional function in the Pinnacle TPS not involving program scripting [7].

Another automatic solution, data-driven knowledge-based planning (KBP), is based on statistical modeling and machine learning approaches attempting to create a predictive model from the library of different patient plans [13]. As a data-driven method, KBP methods can be grouped into two major categories: traditional KBP and deep-learning-based KBP [14]. Traditional KBP utilizes various anatomical and geometrical features to build a predictive model for new cases [15]. RapidPlan (Varian) is an instance of a commercial traditional KBP method that performs a number of retrospective studies [16, 17]. In contrast to traditional KBP using handcrafted features, deep-learning-based KBP methods automatically learn features from raw data by various deep learning network architectures [18, 19].

However, script-based planning is stable but lacks a personalized objective setting, whereas KBP takes patient-specific anatomy into account to generate objectives but relies on a user-dependent optimization process. To take advantage and weaken the defects of the above two automatic planning solutions, some researchers have proposed a hybrid planning solution that combines data-driven KBP methods with commercial script-based methods, known as the traditional commercial KBP method [4] (RapidPlan, Varian) or deep-learning-based method [6], to predict the feasible dose-volume histogram (DVH) for patient-specific objective functions. They then started commercial scripting automatic planning (AutoPlanning, Philips) based on objective functions obtained in the previous step.

However, few studies have fully investigated the advantages of the hybrid solution for the following possible reasons: 1. Most hybrid planning solutions are designed in-house and are therefore not ready for full evaluation; 2. The investigation may require a testing environment with the same TPS and machine model to provide a fair assessment.

In this study, to fully assess the advantages of the hybrid solution, a commercial hybrid automatic planning solution was used. It was compared to manual planning and script-based planning with the same TPS and machine model. By using different initial goal settings, the robustness of these two automatic planning solutions was assessed.

## Methods

### Patients

Overall, 51 rectal cancer patients in our institution were enrolled in this study. These patients were treated between May 2020 and October 2021. All patients were prescribed 5000 cGy in 25 fractions, and the prescription definition was the prescription isodose area covering 95% of the PTV. Each case had CT images scanned in the head supine position. All patients were treated with UIH 506c (United Imaging Healthcare, Shanghai, China) [20].

### Treatment planning

ROIs were manually segmented by oncologists, including clinical target volume (CTV), PTV, bladder, left femur head, right femur head and the external contour.

Each patient had 7 plans in this study, including one clinically accepted manual plan (${plan}_{manual}$), three script-based plans (${plan}_{script}^{easy}$, ${plan}_{script}^{moderate}$, ${plan}_{script}^{hard}$) and three hybrid plans (${plan}_{hybrid}^{easy}$, ${plan}_{hybrid}^{moderate}$, ${plan}_{hybrid}^{hard}$). As a retrospective study, the clinically accepted plans were directly extracted from the TPS database and were generated by expert physicists with at least 3 years of clinical experience and used to treat patients.

All automatic plans were generated with the volumetric-modulated arc therapy (VMAT) technique at UIH TPS (United Imaging Healthcare, Shanghai, China) with the same beam model. Six MV photon dynamic arc beams with one clockwise arc ranging from 0° to 360° were used in each VMAT plan.

### Script-based planning

**Fig. 1:**
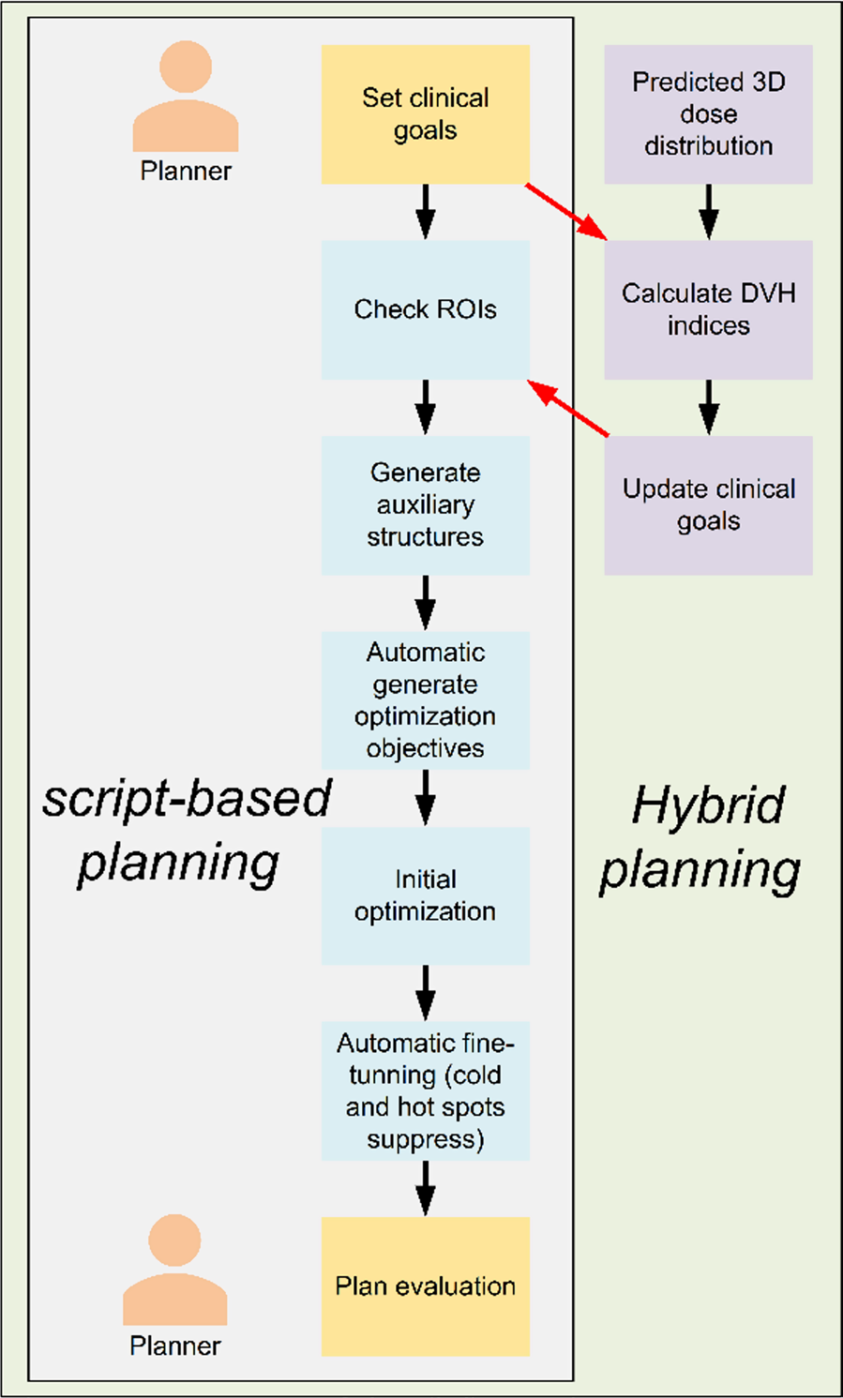
Scheme of script-based planning and hybrid planning

The scheme of script-based planning is shown in Fig. 1. To execute UIH script planning, the planner set a series of clinical goals of dose-volume indices. Compared to optimization objectives in the traditional IMRT process, only a few clinical goals for targets and OARs are needed. The clinical goals of this study are shown in Table 1.

**Table 1:** Three levels of clinical goal setting in this study

Goal setting	Moderate	Hard	Easy
Prescription (D_95_)	5000 (cGy)	5000 (cGy)	5000 (cGy)
Max dose of PTV	5250 (cGy)	5100 (cGy)	5500 (cGy)
Mean dose of bladder	4000 (cGy)	2000 (cGy)	4500 (cGy)
Mean dose of left femur head	1800 (cGy)	900 (cGy)	2700 (cGy)
Mean dose of right femur head	1800 (cGy)	900 (cGy)	2700 (cGy)

According to the vendors’ description, the TPS first checked the required regions of interest, such as target volume, OARs and the external contour, to ensure that there were no improper settings. Then, a series of auxiliary contours were automatically generated, for example, a ring shape structure to shape the dose distribution. Based on the initial optimization settings, TPS automatically generated optimization objectives and respective weights. After initial optimization, regional-based fine-tuning was performed, including cold and hot spot suppression.

### Hybrid planning

Compared to script-based planning, the hybrid planning process could be divided into five steps (Fig. 1): (1) planner sets a series of clinical goals of dose-volume indices; (2) predict achievable 3D dose distribution by a deep-learning model trained with dataset of 190 patient plans in our institution; (3) calculate the dose-volume indices from dose distributions; (4) update the goal of OAR optimization with corresponding dose-volume indices obtained in step (3), while the goals of target will be kept same; (5) run script-based planning.

### Robustness analysis

Script-based planning was dependent on the initial clinical goal setting, while hybrid planning updated these settings. To assess the robustness of this hybrid planning solution for different goal settings, three clinical goal settings—moderate, hard and easy—were created for both the script-based and hybrid automatic plans, as shown in Table 1. The goals of the plan included prescription and max dose of PTV, mean dose of bladder and mean dose of femur head.

### Append auxiliary objective functions

One of the benefits of the 3D dose distribution prediction is that it can be as convenient to append auxiliary without retraining model as the traditional KBP model. To assess the performance of the appending auxiliary objective function, a max dose goal (4200 cGy) on the body contour minus the PTV + 5 mm and a max dose goal (3000 cGy) on the body contour minus the PTV + 15 mm were added, thus simulating a process to improve the conformity of the dose distribution.

To further demonstrate the advantage of hybrid planning solutions, several additional clinical goals were added. The constraint value were determined by the statistical results of ${plan}_{manual}$ shown in Additional file 1: Table S3. Additional clinical goals are the following: for small bowel, V_45Gy_ < 5% and V_15Gy_ < 30%; for both left and right femur head, V_10Gy_ < 75%; for bladder, D_max_ < 52.50 Gy.

### Plan evaluation

A few dose metrics and plan quality metric (PQM) scores were used for plan evaluation.

The dose metrics were as follows: bladder D_15_ and D_50_; left and right femur heads D_25_ and D_40_; PTV D_2_, CI and HI. CI is defined as the product (TV_RI_ / TV) * (TV_RI_ / V_RI_) where TV_RI_ is the target volume covered by the 95% prescription dose, TV is the target volume and V_RI_ is the volume of the 95% prescription isodose. A CI closer to 1 indicates better conformity in the PTV, HI is defined as the ratio (D_2_-D_98_)/D_prescription_ [10], and HI closer to 0 indicates better homogeneity.

As shown in Table 2, a PQM scoring procedure with 9 metrics was defined for the plans. The PQM score of each metric was calculated using the quantity and the PQM value range set for the metric [21].

**Table 2:** Detailed setting of PQM scoring

ROI	Index	Metric		PQM value	
		Lower limit	Upper limit	Minimum	Maximum
PTV	D_2_	5250	5500	0	10
	HI	0	0.15	0	10
	CI	0.8	0.9	0	10
Bladder	D_15_	4000	5250	0	10
	D_50_	2500	4500	0	10
Left femur head	D_25_	2000	3000	0	10
	D_40_	1250	2500	0	10
Right femur head	D_25_	2000	3000	0	10
	D_40_	1250	2500	0	10

A paired t-test or Wilcoxon signed rank test (R version 4.1.2) was used to compare the differences among the three planning methods. A *p value* less than 0.05 was considered statistically significant.

## Results

### Patient characteristics

A total of 51 patients were enrolled in this study. Patient characteristics are shown in Table 3.

**Table 3:** Patient characteristics

Characteristics	Value
Total number of patients	51
*Age*	
< 40 years	4
40–50 years	12
50–60 years	10
60–70 years	20
> 70 years	5
*Sex*	
Male	38
Female	13
*Distance to the Anal Verge*	
< 4 cm	18
4–6 cm	14
6–8 cm	12
> 8 cm	4
Missing values	3

### Comparison to manual plan

**Fig. 2:**
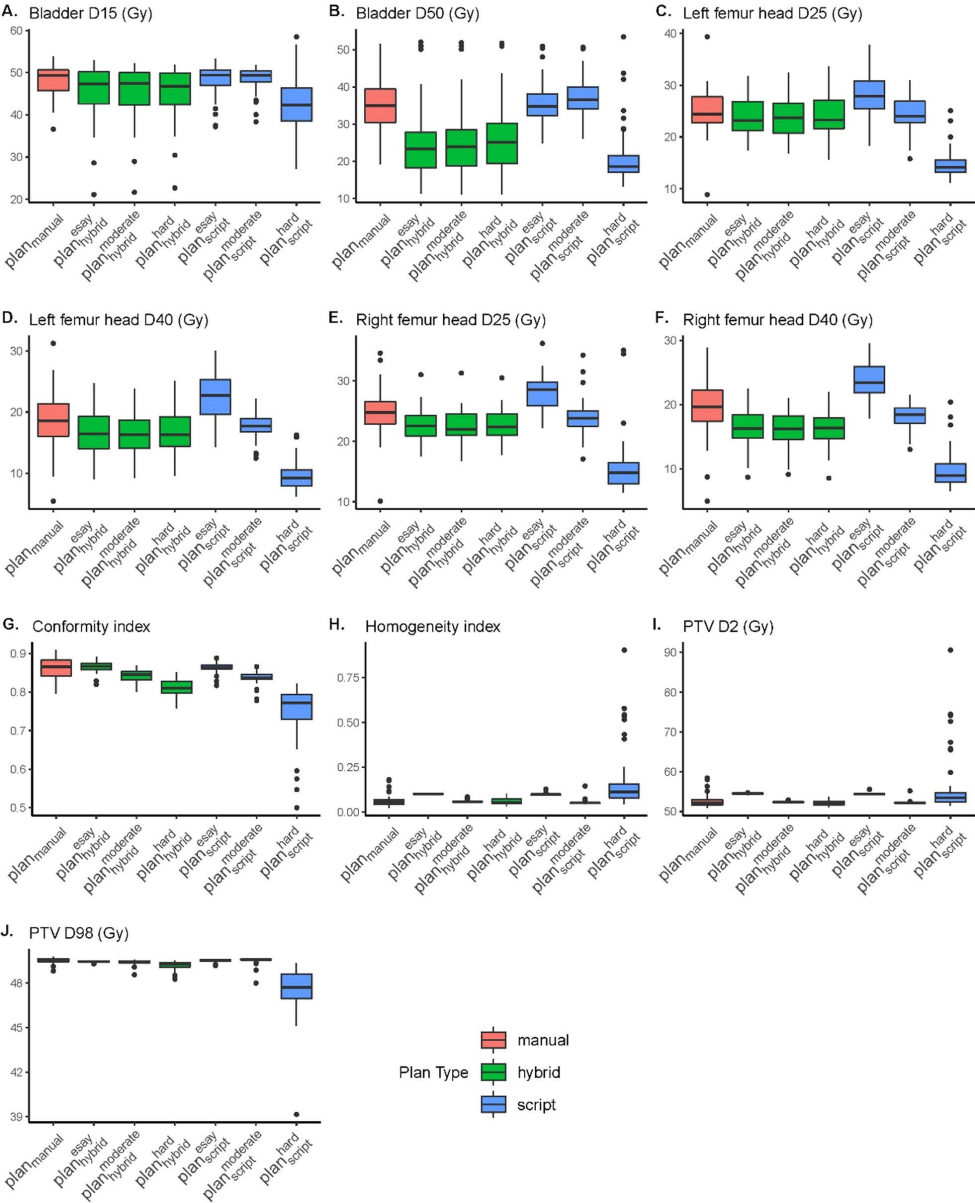
Dose comparison of ${plan}_{manual}$, ${plan}_{hybrid}^{easy}$, ${plan}_{hybrid}^{moderate}$, ${plan}_{hybrid}^{hard}$, ${plan}_{script}^{easy}$, ${plan}_{script}^{moderate}$ and ${plan}_{script}^{hard}$.** A** Bladder D15.** B** Bladder D50.** C** Left femur head D25.** D** Left femur head D40.** E** Right femur head D25.** F** Right femur head D40.** G** Conformity index.** H** Homogeneity index.** I** PTV D2.** J** PTV D98

Figure 2 presents the dose comparison of ${plan}_{manual}$, ${plan}_{hybrid}^{easy}$, ${plan}_{hybrid}^{moderate}$, ${plan}_{hybrid}^{hard}$, ${plan}_{script}^{easy}$, ${plan}_{script}^{moderate}$ and ${plan}_{script}^{hard}$.

Compared to ${plan}_{manual}$, ${plan}_{hybrid}^{easy}$ significantly reduced the OAR dose (*p* < 0.05) for all dose-volume indices, except D_25_ of the left femur head (*p* = 0.07, paired t-test). However, HI was increased as expected, since a loose PTV constraint was used. For moderate and hard constraints, the hybrid strategy obtained similar OAR dose-volume indices. Compared to ${plan}_{manual}$, the OAR dose for all dose-volume indices was also significantly reduced (*p* < 0.05), except D_25_ of the left femur head. With stricter PTV constraints, no significant difference for HI was found (*p* = 0.37 and *p* = 0.70 for moderate and hard settings, respectively). The CI for the hybrid strategy was lower than that for the manual plan. The detailed data can be found in Additional file 1: Table S1.

For script-based planning, ${plan}_{script}^{easy}$ significantly increased the OAR dose for the femur head (*p* < 0.05). Meanwhile, the PTV D_2_, D_5_ and HI were higher than those of the manual plan. With a moderate clinical goal setting, the script plan provided results similar to manual planning. For the hard clinical goals setting, although the OAR dose was reduced by stricter clinical goals setting, the PTV HI was much worse than the manual plan (HI = 0.17 vs. HI = 0.06, for ${plan}_{script}^{hard}$ and ${plan}_{manual}$, *p* < 0.001). The detailed data can be found in Additional file 1: Table S2.

Figure 2 also shows a significant dose-volume index variation between different clinical goal settings for script-based planning; for example, the D_40_ of the left femur was 27.74 Gy, 24.50 Gy and 14.77 Gy for easy, moderate and hard clinical goal settings, respectively. However, the hybrid method remained relatively stable for OAR dose-volume indices. Because both strategies used different PTV goal settings, the PTV dose-volume indices varied for both strategies. However, the hybrid strategy was still relatively more stable than the script-based strategy. For example, the CI was decreased from 0.87 to 0.81 for hybrid-based planning. In contrast, the CI was decreased from 0.86 to 0.75 for script-based planning.

### Append auxiliary ROI

**Fig. 3:**
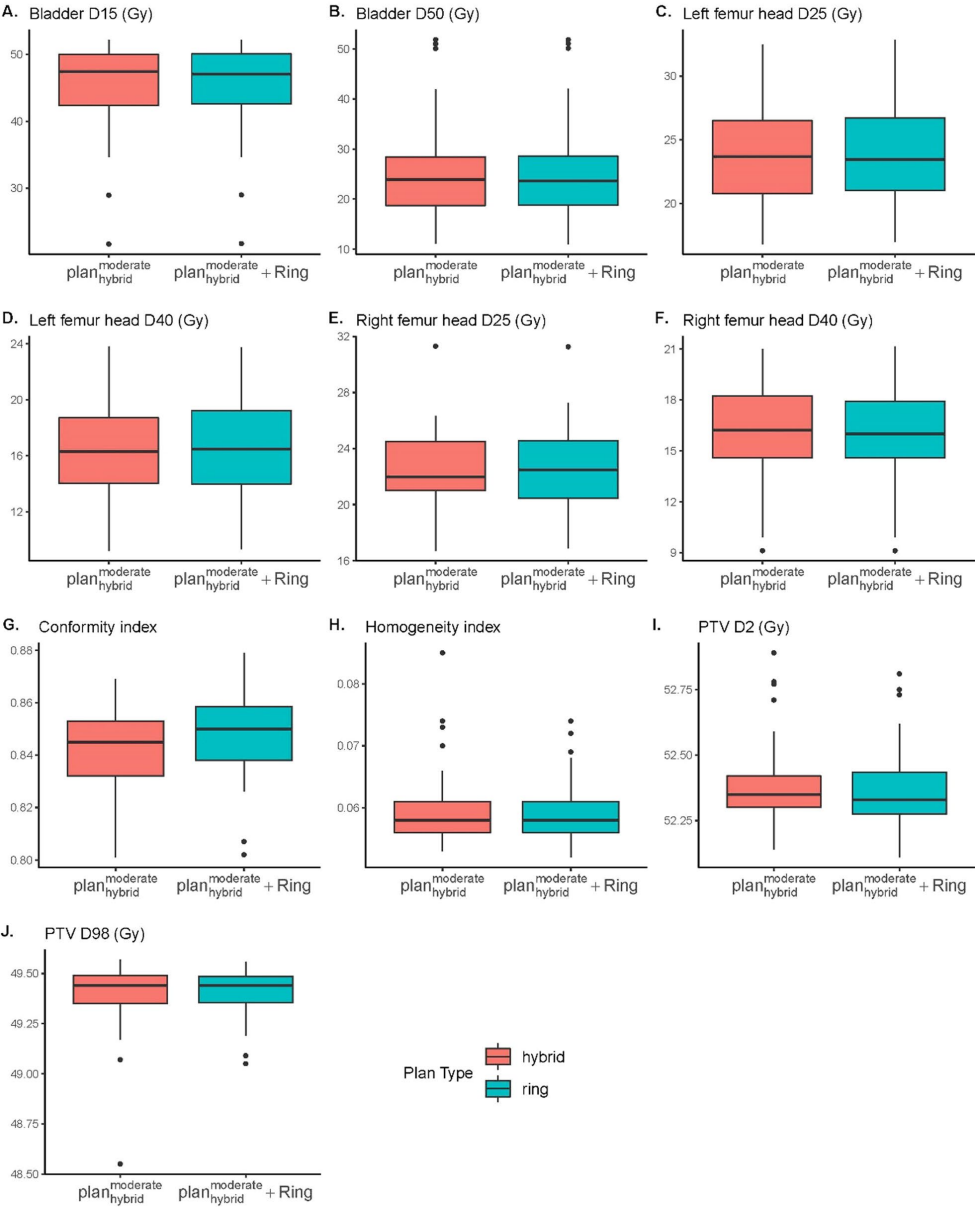
Dose comparison of ${plan}_{hybrid}^{moderate}$ and ${plan}_{hybrid}^{moderate}$ with additional auxiliary ROI.** A** Bladder D15.** B** Bladder D50.** C** Left femur head D25.** D** Left femur head D40.** E** Right femur head D25.** F** Right femur head D40.** G** Conformity index.** H** Homogeneity index.** I** PTV D2.** J** PTV D98

Figure 3 shows the results of appending the auxiliary ROI. By adding a ring shape auxiliary ROI, the conformity index was significantly increased from 0.842 to 0.847 (*p* < 0.001, paired Wilcox test).

### Additional clinical goals

**Fig. 4:**
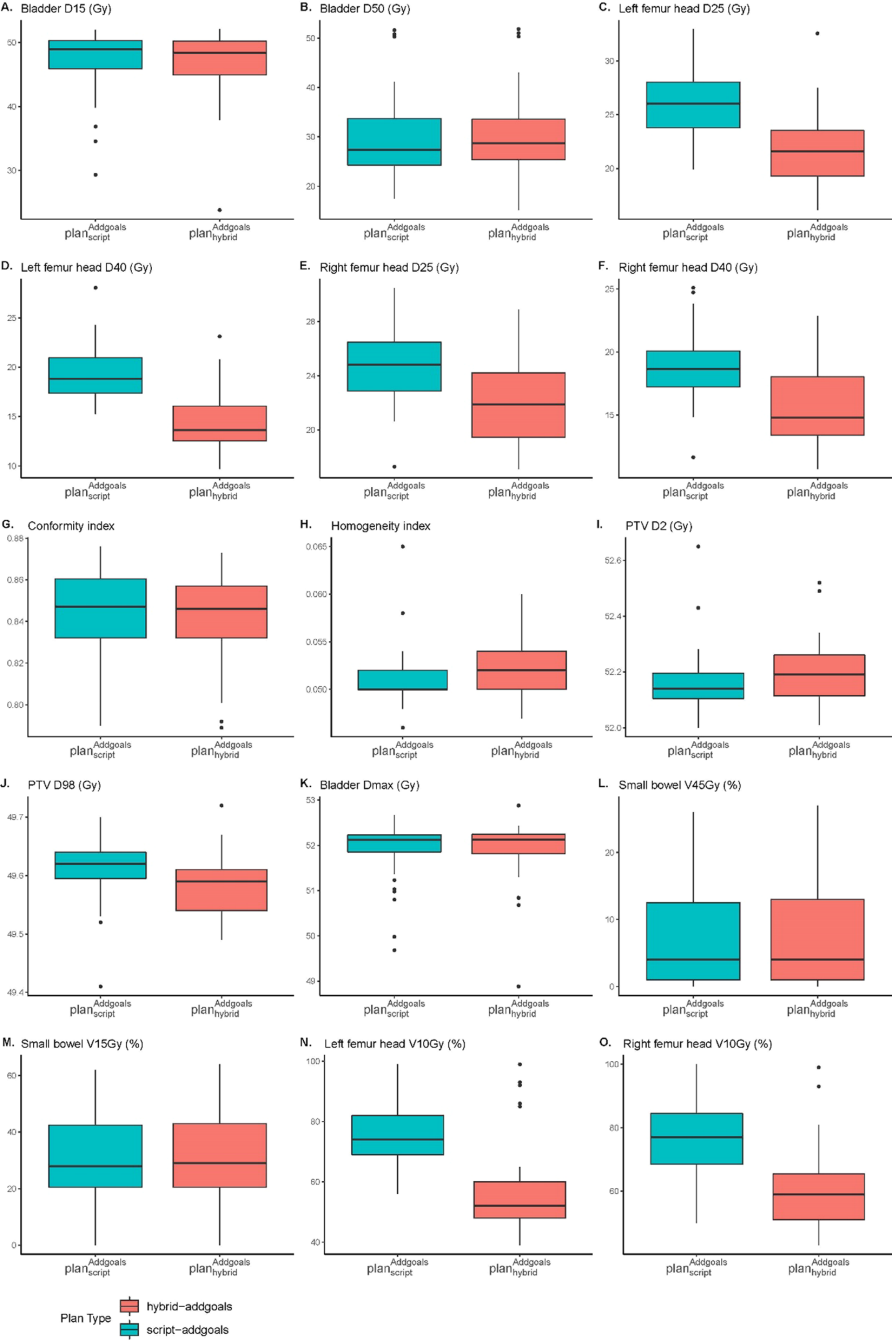
Dose comparison between ${plan}_{script}^{moderate}$ with additional clinical goals and ${plan}_{hybrid}^{moderate}$ with additional clinical goals.** A** Bladder D15.** B** Bladder D50.** C** Left femur head D25.** D** Left femur head D40.** E** Right femur head D25.** F** Right femur head D40.** G** Conformity index.** H** Homogeneity index.** I** PTV D2.** J** PTV D98.** K** Bladder Dmax.** L** Small bowel V45.** M** Small bowel V15.** N** Left femur head V10.** O** Right femur head V10

Figure 4 shows the comparing results between ${plan}_{hybrid}^{moderate}$ with additional clinical goals and ${plan}_{script}^{moderate}$ with additional clinical goals. By adding more clinical goals, hybrid planning pays more attention to users’ interested region like small bowel and femur head in this case, which contributes to lower dose in femur head and small bowel while sacrificing dose distribution of PTV and the area around PTV. Comparing with ${plan}_{script}^{moderate}$ adding clinical goals, ${plan}_{hybrid}^{moderate}$ with additional clinical goal decreased dose of femur head significantly (*p* < 0.05).

### Time evaluation

The time for ${plan}_{hybrid}^{easy}$ and ${plan}_{hybrid}^{moderate}$ is average 4 min. ${Plan}_{hybrid}^{hard}$ costs average 5 min. The absolute value of difference costing time between hybrid plan and script-based plan is average 10 s. In comparison, ${plan}_{manual}$ takes 30–40 min.PQM scoring results.

Table 4 shows the details of the PQM score and data distribution. For all three clinical goal settings, statistical comparison of metrics in PQM between hybrid planning and script-based planning showed significant differences, except for D_25_ of the left femur head and PTV D_2_ for the moderate goal setting. Hybrid planning obtained a higher score of PTV CI. For moderate and easy goal settings, OARs fared better in hybrid planning. For hard goal settings, script-based planning performed better in OAR sparing but sacrificed all three metrics (D_2_, HI, CI) of the PTV. In particular, there were 11/51 s${plan}_{script}^{hard}$ whose D_2_ exceeded the maximum acceptable value (5500 cGy), and the mean $\pm$ standard deviation was 6705 $\pm$ 1064 (cGy). These plans were not clinically acceptable.

**Table 4:** Comparison of PQM scores between hybrid plans and script-based plans for three goal settings

ROI	Index	Moderate HP vs. SP	*p-value*	Easy HP vs. SP	*p-value*	Hard HP vs. SP	*p-value*
PTV	D2	9.90 vs. 9.80	0.14	2.02 vs. 2.44	< 0.001*	9.46 vs. 5.67	< 0.001*
	HI	6.03 vs. 6.39	< 0.001*	3.24 vs. 3.48	< 0.001*	5.99 vs. 2.70	< 0.001*
	CI	4.26 vs. 3.80	< 0.001*	6.65 vs. 6.36	0.006*	1.59 vs. 0.21	< 0.001*
Bladder	D15	4.98 vs. 3.21	< 0.001*	4.95 vs. 3.29	< 0.001*	5.09 vs. 7.06	< 0.001*
	D50	8.38 vs. 4.13	< 0.001*	8.49 vs. 4.78	< 0.001*	8.13 vs. 9.19	< 0.001*
Left femur head	D25	6.00 vs. 5.38	0.06	6.03 vs. 2.91	< 0.001*	5.65 vs. 9.84	< 0.001*
	D40	6.62 vs. 5.76	< 0.001*	6.49 vs. 2.55	< 0.001*	6.48 vs. 9.82	< 0.001*
Right femur head	D25	7.24 vs. 6.02	< 0.001*	7.20 vs. 2.36	< 0.001*	7.07 vs. 9.55	< 0.001*
	D40	6.86 vs. 5.34	< 0.001*	6.87 vs. 1.56	< 0.001*	6.89 vs. 9.67	< 0.001*
Summary		65.24 vs. 53.04	< 0.001*	56.89 vs. 33.03	< 0.001*	61.44 vs. 70.78#	< 0.001*

*Note*: HP represents hybrid planning, and SP represents scripted-based planning

^*^Statistically significant

^#^ The score should not be interpreted as better plan quality: there were 11/51 plans whose D2 exceeded the maximum acceptable value (5500 cGy)

## Discussion

This study evaluated a hybrid automatic planning solution by comparing it with manual planning and script-based planning for rectal cancer. The results showed that the hybrid solution could better spare OARs and increase the robustness for automatic planning.

The hybrid planning performed significantly better than manual planning in OAR sparing (Additional file 1: Table S1), except for left femur head D_25_, which showed no significant difference. This result is similar to the conclusion proposed by Hansen et al. [22], who concluded that, on average, automated planning reduces the OAR dose compared to manual planning.

A comparison between hybrid planning and script-based planning showed that hybrid planning could be more robust and further indicated that it is beneficial to use a more personalized goal setting for automatic planning, which was also reported by Fan et al. [3], Ling et al. [4] and Xia et al. [21].

For easy goal setting, script-based planning resulted in little decline in the high dose of PTV but worsened OAR sparing (Additional file 1: Table S2), which contributed to the low PQM score of 56.89, which was lower than 33.03 in hybrid planning (Table 4).

**Fig. 5:**
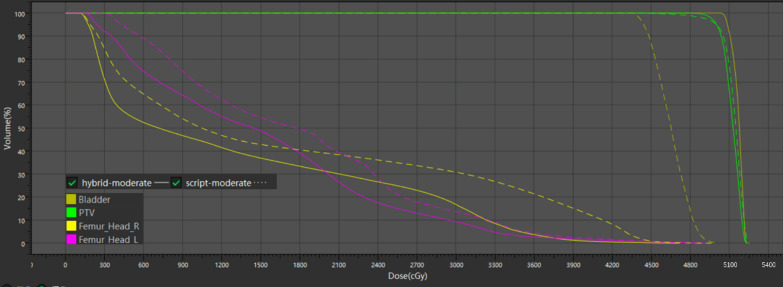
DVH of one patient. The solid line represents hybrid planning, and the dashed line represents script-based planning

From the total PQM perspective, the advantages of hybrid planning for moderate goal setting were not as obvious as those for easy goal setting (65.24 for hybrid planning and 53.04 for script-based planning), but the improvement in robustness could be found in specific cases. Figure 5 shows the DVH results for a patient whose bladder largely overlapped with the PTV. Hybrid planning automatically lost the constraint of the bladder.

For hard goal settings, while script-based planning obtained higher PQM scores than the hybrid, it could not be concluded that ${plan}_{script}^{hard}$ performed better because many plans had clinically unacceptable PTV D_2_. This means that script-based solutions may give too many weights in OAR constraints if planners do not provide appropriate clinical goals.

The hybrid planning solution in this study used a deep-learning-based method to predict a feasible DVH for patient-specific objective functions and then started scripting automatic planning based on objective functions obtained in the previous step. Obtaining objective functions through predicted dose distribution provides the convenience to add clinical goals. Different from generating plans based on patient-specific DVH objectives, efforts in voxel-based optimization strategies were also made: predicting the 3D distribution and then exporting it into an open-source [3] or commercial TPS [23, 24] for script-based automatic optimization. While 3D dose distribution reserves more spatial information than DVH, it could lead to some other problems, for example, hot or cold spots in targets in those imperfect dose distribution predictions due to possible violations of laws of physics [23]. There were still some limitations in this study. First, the dataset used for deep learning model training might not have been the optimal plan, which could have contributed to the suboptimal predicted dose metric values. Second, the beam setting of manual planning was not taken into account, which may have had a considerable impact on the results. Investigation of training data selection and plan parameters of manual planning will continue.

## Conclusions

The hybrid planning solution was manual-planning comparable and robust. Comparisons of PQM score and dose metrics between hybrid planning and script-based planning showed that hybrid planning was more robust.

## Supplementary Information

**Additional file 1**. Table S1. Statistical comparison among manual plans and hybrid plans for moderate, hard and easy clinical goal settings. Table S2. Statistical comparison among manual plans and script plans for moderate, hard and easy clinical goal settings. Table S3. Statistical results of planmanual for added goals setting.

## Data Availability

The datasets used and/or analyzed during the current study are available from the corresponding author on reasonable request.

## Abbreviations

KBP: Knowledge-based planning
VMAT: Volumetric-modulated arc therapy
PTV: Planning target volume
OAR: Organ at risk
PQM: Plan quality metric
IMRT: Intensity-modulated radiation therapy
DVH: Dose-volume histogram
CI: Conformity index
HI: Homogeneity index
CTV: Clinical target volume
