# Supplementary material for: Evaluation of a hybrid automatic planning solution for rectal cancer
Source: Radiat Oncol. 2022 Oct 13;17:166. doi: 10.1186/s13014-022-02129-9 (PMC9563136; doi:10.1186/s13014-022-02129-9)
Supplement: Supplementary file 1 — Additional file 1. Table S1. Statistical comparison among manual plans and hybrid plans for moderate, hard and easy clinical goal settings. Table S2. Statistical comparison among manual plans and script plans for moderate, hard and easy clinical goal settings. Table S3. Statistical results of planmanual for added goals setting. [file 13014_2022_2129_MOESM1_ESM.docx]

# Supplementary Material

**Supplementary Table S1**. Statistical comparison among manual plans and hybrid plans for moderate, hard and easy clinical goal settings.

| OAR | Index | ${plan}_{manual}$ | ${plan}_{script}^{moderate}$ | ${plan}_{manual}$ vs*.*  ${plan}_{script}^{moderate}$ | ${plan}_{script}^{easy}$ | ${plan}_{manual}$  vs*.*  ${plan}_{script}^{easy}$ | ${plan}_{hybrid}^{hard}$ | ${plan}_{manual}$  vs.  ${plan}_{script}^{hard}$ |
| --- | --- | --- | --- | --- | --- | --- | --- | --- |
| Bladder | D15, Gy | 47.88$\pm$3.82 | 45.28$\pm$6.25 | < 0.001* | 45.30$\pm$6.46 | < 0.001* | 45.18$\pm$6.09 | < 0.001* |
|  | D50, Gy | 34.77$\pm$7.58 | 24.94$\pm$9.74 | < 0.001* | 24.49$\pm$9.68 | < 0.001* | 25.90$\pm$9.74 | < 0.001* |
| Left femur head | D25, Gy | 25.02$\pm$4.32 | 23.93$\pm$3.60 | 0.10 | 23.87$\pm$3.43 | 0.08 | 24.25$\pm$4.12 | 0.26 |
|  | D40, Gy | 18.81$\pm$4.63 | 16.65$\pm$3.24 | 0.002* | 16.81$\pm$3.34 | 0.003* | 16.84$\pm$3.50 | 0.005* |
| Right femur head | D25, Gy | 24.86$\pm$4.07 | 22.53$\pm$2.77 | < 0.001* | 22.60$\pm$2.73 | < 0.001* | 22.73$\pm$2.72 | 0.001* |
|  | D40, Gy | 19.61$\pm$4.51 | 16.27$\pm$2.73 | < 0.001* | 16.26$\pm$2.77 | < 0.001* | 16.26$\pm$2.66 | < 0.001* |
| PTV | D2, Gy | 52.63$\pm$1.52 | 52.38$\pm$0.15 | 0.80 | 54.50$\pm$0.08 | < 0.001* | 52.18$\pm$0.61 | 0.25 |
|  | D5, Gy | 52.44$\pm$1.46 | 52.30$\pm$0.16 | 0.35 | 54.35$\pm$0.09 | < 0.001* | 52.10$\pm$0.60 | 0.56 |
|  | D98, Gy | 49.50$\pm$0.18 | 49.40$\pm$0.16 | 0.002* | 49.43$\pm$0.06 | < 0.001* | 49.17$\pm$0.28 | < 0.001* |
|  | HI | 0.06$\pm$0.03 | 0.06$\pm$0.01 | 0.37 | 0.1$\pm$0.0 | < 0.001* | 0.06$\pm$0.02 | 0.70 |
|  | CI | 0.86$\pm$0.03 | 0.84$\pm$0.02 | < 0.001* | 0.87$\pm$0.01 | 0.31 | 0.81$\pm$0.02 | < 0.001* |

**Note:** *Statistically significant.

**Supplementary Table S2**. Statistical comparison among manual plans and script plans for moderate, hard and easy clinical goal settings.

| OAR | Index | ${plan}_{manual}$ | ${plan}_{script}^{moderate}$ | ${plan}_{manual}$ vs*.*  ${plan}_{script}^{moderate}$ | ${plan}_{script}^{easy}$ | ${plan}_{manual}$  vs*.*  ${plan}_{script}^{easy}$ | ${plan}_{script}^{hard}$ | ${plan}_{manual}$  vs.  ${plan}_{script}^{hard}$ |
| --- | --- | --- | --- | --- | --- | --- | --- | --- |
| Bladder | D15, Gy | 47.88$\pm$3.82 | 48.45±2.84 | 0.20 | 48.30±3.57 | 0.15 | 42.77±6.15 | < 0.001* |
|  | D50, Gy | 34.77$\pm$7.58 | 36.99±5.09 | 0.017* | 35.71±5.54 | 0.48 | 21.14±7.76 | < 0.001* |
| Left femur head | D25, Gy | 25.02$\pm$4.32 | 24.50±3.00 | 0.36 | 27.74±4.18 | <0.001* | 14.77±2.63 | < 0.001* |
|  | D40, Gy | 18.81$\pm$4.63 | 17.80±2.00 | 0.15 | 22.52±4.19 | <0.001* | 9.61±2.33 | < 0.001* |
| Right femur head | D25, Gy | 24.86$\pm$4.07 | 24.02±2.83 | 0.20 | 28.00±2.88 | < 0.001* | 15.60±4.60 | < 0.001* |
|  | D40, Gy | 19.61$\pm$4.51 | 18.33±1.84 | 0.032* | 23.84±3.08 | < 0.001* | 9.78±2.82 | < 0.001* |
| PTV | D2, Gy | 52.63$\pm$1.52 | 52.23±0.44 | 0.24 | 54.40±0.20 | < 0.001* | 56.06±7.57 | < 0.001* |
|  | D5, Gy | 52.44$\pm$1.46 | 52.14±0.43 | 0.72 | 54.25±0.19 | < 0.001* | 55.67±6.77 | < 0.001* |
|  | D98, Gy | 49.50$\pm$0.18 | 49.53±0.25 | 0.09 | 49.51±0.07 | 0.79 | 47.56±1.60 | < 0.001* |
|  | HI | 0.06$\pm$0.03 | 0.05±0.01 | 0.18 | 0.1±0 | < 0.001* | 0.17±0.17 | < 0.001* |
|  | CI | 0.86$\pm$0.03 | 0.84±0.02 | < 0.001* | 0.86±0.01 | 0.75 | 0.75±0.07 | < 0.001* |

**Supplementary Table S3.** Statistical results of ${plan}_{manual}$ for added goals setting.

|  | Small Bowel |  | Left  femur head | Right  femur head | Bladder |
| --- | --- | --- | --- | --- | --- |
|  | V_45Gy_ (%) | V_15Gy_ (%) | V_10Gy_ (%) | V_10Gy_ (%) | D_max_ (Gy) |
| Mean | 6 | 31 | 75 | 74 | 52.40 |
| Std.Dev. | 6 | 16 | 18 | 18 | 1.47 |
| Median | 4 | 28 | 76 | 72 | 52.16 |
| Range | [0,21] | [0,62] | [22,100] | [25,100] | [49.25,58.69] |
